# *Sedum takesimense* Protects PC12 Cells against Corticosterone-Induced Neurotoxicity by Inhibiting Neural Apoptosis

**DOI:** 10.3390/nu12123713

**Published:** 2020-11-30

**Authors:** Hea-Yeon Yun, Yoonhwa Jeong

**Affiliations:** 1 Research Center for Industrialization of Natural Nutraceuticals, Dankook University, Cheonan 31116, Korea; yunheayeon@gmail.com; 2 Department of Food Science and Nutrition, Dankook University, Cheonan 31116, Korea

**Keywords:** apoptosis, ER stress, mitochondrial dysfunction, PC12 cells, *Sedum takesimense*

## Abstract

Neuronal cell death induced by chronic stress in the central nervous system is a cause of neurological dysfunction. We investigated the neuroprotective potential of a water extract of *S. takesimense* (WEST) against corticosterone-induced apoptosis in PC12 cells and the possible underlying mechanisms. Cells were pretreated with 50 µg/mL of WEST to evaluate its neuroprotective effect based on endoplasmic reticulum (ER) stress inhibition and mitochondrial function improvement. Pretreatment with WEST prevented corticosterone-induced injury in PC12 cells, resulting in increased cell survival, decreased lactate dehydrogenase (LDH) release, and potent apoptosis inhibition by a reduction in apoptotic nuclei demonstrated by Hoechst 33342 and propidium iodide (PI) double staining, and TUNEL staining. WEST strongly attenuated calcium (Ca^2+^) elevation, inducing the closure of mitochondrial permeability transition pores (mPTPs), which were opened by corticosterone. It also stabilized mitochondrial membrane potential (MMP) loss and inhibited the corticosterone-induced decrease in adenosine triphosphate (ATP) levels. Furthermore, the increased reactive oxygen species (ROS) production induced by corticosterone was prevented in PC12 cells treated with WEST. WEST also downregulated the expression of glucose-regulated protein 78 (GRP78), growth arrest- and DNA damage-inducible gene 153 (GADD153), the pro-apoptotic protein Bcl-2-associated X (Bax), cytochrome c, cysteine-aspartic protease (caspase)-9, and caspase-3, and upregulated the expression of the anti-apoptotic protein B-cell lymphoma 2 (Bcl-2). Thus, WEST exerts a neuroprotective effect by inhibiting the apoptosis pathway in ER stress and the mitochondrial dysfunction induced by corticosterone. These results demonstrate that WEST reduces neuronal damage from the neurotoxicity caused by chronic stress.

## 1. Introduction

When a stressor (psychological or physical) is sensed in the body, a signal is sent to the hypothalamus, activating the hypothalamic–pituitary–adrenal (HPA) axis [1]. This leads to the synthesis of glucocorticoids in the adrenal cortex [2]. Glucocorticoids play an essential role in the regulation of a variety of processes, including metabolism and immune function [3]. Glucocorticoid secretion and the level of glucocorticoids in the blood are regulated via the negative feedback suppression of the HPA axis [4,5]. However, sustained exposure to high concentrations of glucocorticoids in the blood, due to extreme or chronic stress, can cause dysfunction of the HPA axis and negative feedback mechanisms, resulting in the excessive secretion of glucocorticoids, which causes damage to the nervous system [6,7,8].

It is reported that continuous exposure to high concentrations of glucocorticoids causes DNA damage in hippocampal nerve cells that encode and recall memories, eventually leading to apoptosis in these cells [9,10,11]. Glucocorticoid-induced apoptosis is a cause of neurological dysfunction, including memory loss, learning disabilities, and cognitive impairment, and can also trigger mental disorders such as anxiety and depression [12,13,14,15,16]. Thus, if the neurons can be protected from glucocorticoid-induced damage, neurological dysfunction due to chronic stress may be reduced.

Although benzodiazepines are used to treat stress-related nervous system disorders, the long-term use or combination of drugs can cause severe side effects, including drug addiction, drowsiness, vertigo, mental confusion, muscle weakness, and movement disorders [17]. Therefore, it is necessary to find natural substances that are safe for long-term use without causing severe side effects.

*Sedum* is a large genus in the family Crassulaceae, and its phytochemical composition has been widely reported [18,19,20]. *Sedum* plants have been used in traditional medicine for their antitumor, anti-inflammation, vasodilation, and wound-healing properties in various countries for a long time [18,19,20,21,22]. *S. takesimense* is a medicinal plant species endemic to Ulleung Island. Cell studies have demonstrated anti-inflammatory effects for *S. takesimense* [23], and Vu et al. (2013) identified antibacterial compounds from *S. takesimense*, including eight antibacterial gallotannins such as gallic acid, methyl gallate, and 4,6-di-*O*-galloylarbutin by electrospray ionization mass spectrometry and proton nuclear magnetic resonance spectroscopy [22]. Antioxidant compounds have also been identified in *S. takesimense*, including phenolic constituents such as 2,6-di-*O*-galloylarbutin, gossypetin-8-*O*-beta-d-xylopyranoside, and 1-(4-hydroxyphenyl)-2-(3,5-dihydroxyphenyl)-2-hydroxyethanone, by spectroscopic analyses (IR, UV, NMR, and HR-MS) and chemical degradation [24] (Table 1). However, there have been no studies regarding its neuroprotective effect on glucocorticoid-induced neuronal damage and the potential underlying mechanisms.

In the present study, we investigated the neuroprotective effect of water extract of *S. takesimense* (WEST) against stress inducers and their underlying mechanisms. We used the PC12 cell line, which is derived from a rat pheochromocytoma and expresses high levels of glucocorticoid receptors, and which is widely used in nerve injury studies, among others [25,26,27]. Corticosterone, the major glucocorticoid in rodents [9], was used to induce cellular stress in this study.

We demonstrated the neuroprotective effect of WEST against corticosterone-induced apoptosis in PC12 cells and discuss whether the cytoprotective effects of WEST occur via the inhibition of ER stress and improved mitochondrial dysfunction.

## 2. Materials and Methods

### 2.1. Preparation of WEST

*S. takesimense* was obtained from Ulleung County, North Gyeongsang Province, Korea, in January 2018. Leaves of *S. takesimense* were dried, and the dried leaves (10 g) were extracted with distilled water (1:10 (wt/vol)) twice (each time for 90 min) in a reflux extractor. The water extracts were combined and filtered through an 8 µm-pore-size filter paper (Whatman™ Grade 2, GE Healthcare, Sheffield, UK) and then evaporated under reduced pressure using a rotary evaporator (N-1000S, Tokyo rikakikai Co., Tokyo, Japan). The concentrated extracts were freeze-dried using a freeze dryer, FD8505 (Ilshin Lab Co., Ltd., Seoul, Korea).

### 2.2. Cell Culture and Treatment

PC12 cells were purchased from the Korean Cell Line Bank (Seoul, Korea) and grown in RPMI 1640 medium supplemented with 10% (*v*/*v*) fetal bovine serum (FBS), 100 U/mL of penicillin, and 100 µg/mL of streptomycin in a humidified atmosphere of 5% CO_2_ and 95% air at 37 °C. Twenty-four hours after the cells were seeded, the medium was refreshed with RPMI 1640 containing 1% (*v*/*v*) FBS and the same antibiotics as described above.

To investigate the cytoprotective effect of WEST, the cells were divided into four groups: non-treated control (CON), 200 µM corticosterone treatment only (CORT), 50 µg/mL WEST pretreatment plus 200 µM corticosterone (WEST + CORT), and 50 µg/mL WEST treatment only (WEST). Twenty-four hours after seeding the cells, WEST was incubated for 2 h before the treatment with corticosterone, and then, the cells were co-incubated with WEST and corticosterone for 24 h.

### 2.3. Cell Viability Assay

Cell viability was evaluated using the EZ-CYTOX kit (Daeil Lab, Seoul, Korea) according to the manufacturer’s instructions. Briefly, cells (5 × 10^4^ cells/well) were seeded in 96-well plates, and after 24 h of treatment, the cells were co-incubated with 20 µL of EZ-Cytox solution in 200 µL of total cell volume for 2 h at 37 °C in darkness. The absorbance was measured at a wavelength of 450 nm using a spectrophotometer (SpectraMax M2 microplate reader, Molecular Devices, Sunnyvale, CA, USA).

### 2.4. LDH Leakage Assay

To determine the intensity of cell injury, the activity of lactate dehydrogenase (LDH) released from cells was measured using an EZ-LDH kit (Daeil Lab, Seoul, Korea). Cells were seeded in 96-well plates. At the end of the treatment, the supernatant was collected and reacted with the LDH reaction mixture following the manufacturer’s protocol. After 1 h at room temperature in darkness, the absorbance of the samples was measured at 450 nm using a spectrophotometer (SpectraMax M2 microplate reader, Molecular Devices, Sunnyvale, CA, USA).

### 2.5. Hoechst 33342 and PI Double Staining

Chromatin condensation was analyzed by nucleus staining with Hoechst 33342 and PI double staining. Cells were plated on 18 mm coverslips. After the indicated treatment, the fixed cells were stained with 8.1 µM Hoechst 33342 solution (Thermo Fisher Scientific, Waltham, MA, USA) for 15 min at room temperature and then co-stained with 1.5 µM PI solution (Thermo Fisher Scientific, Waltham, MA, USA) for another 15 min. The cells were visualized by fluorescence microscopy (ZEISS, Jena, Germany), and the apoptotic nuclei were counted in five randomly selected fields in each group in three experiments. The data are expressed as a percentage of the total number of nuclei counted.

### 2.6. TUNEL Staining

Internucleosomal DNA fragmentation was detected using the terminal deoxynucleotidyl transferase (TdT)-mediated dUTP nick end labeling (TUNEL) kit (Promega, Madison, WI, USA). In short, cells were cultured on 18 mm coverslips. After the indicated treatment, the fixed cells were incubated with TUNEL reaction mixture for 1 h at 37 °C according to the manufacturer’s protocol and then viewed under a fluorescence microscope (ZEISS, Jena, Germany). TUNEL-positive nuclei were counted in five randomly chosen fields per coverslip three times; then, the apoptotic percentage was calculated by comparing the TUNEL-positive counts with the total cell nuclei, determined by Hoechst 33342 counterstaining.

### 2.7. Intracellular ROS Level Assay

Intracellular reactive oxygen species (ROS) levels were measured using 2′,7′-dichlorofluorescein diacetate (DCF-DA) (Sigma-Aldrich, Inc., St. Louis, MO, USA). Briefly, cells were seeded into a 96-well black plate. At the end of treatment, the culture medium was removed, and the cells were incubated with 10 µM DCF-DA for 1 h at 37 °C. The fluorescence of the DCF was measured with a fluorescence spectrophotometer (SpectraMax M2 microplate reader, Molecular Devices, Sunnyvale, CA, USA) at excitation and emission wavelengths of 485 and 535 nm, respectively.

### 2.8. Intracellular Ca^2+^ Level Assay

Intracellular Ca^2+^ levels were measured using the Fura-2/AM (Thermo Fisher Scientific, Waltham, MA, USA). Briefly, cells were seeded in a 96-well black plate. After the indicated treatment, the cells were collected and co-incubated with a 5 µM Fura-2/AM working solution for 1 h at 37 °C. The fluorescence intensity was measured using a fluorescence spectrophotometer at excitation wavelengths of 340 nm (Ca^2+^-bound form) and 380 nm (Ca^2+^-unbound form), with an emission wavelength of 510 nm. The intracellular Ca^2+^ level was reflected by the ratio of fluorescence (F_340_/F_380_).

### 2.9. Detection of mPTP Opening

The mitochondrial permeability transition pore (mPTP) opening of mitochondria was detected using a mitochondrial transition pore assay kit using the calcein–cobalt quenching method (Thermo Fisher Scientific, Waltham, MA, USA). Briefly, cells were plated on 18 mm coverslips. After treatment, the fixed cells were labeled with the labeling solution (including 1 µM calcein AM and 1 mM cobalt chloride) for 15 min at 37 °C according to the kit manufacturer’s instructions, and visualized by fluorescence microscopy (ZEISS, Jena, Germany).

### 2.10. Measurement of MMP

Changes in the mitochondrial membrane potential (MMP) were assessed with a mitochondrial membrane potential kit using the JC-10 dye (Sigma-Aldrich, St. Louis, MO, USA). Briefly, cells were seeded into a 96-well black plate. After the indicated treatment, the cells were co-incubated with the JC-10 solution for 1 h at 37 °C according to the kit manufacturer’s instructions. Monomeric JC-10 green fluorescence (λ_ex_ = 490/λ_em_ = 525 nm) and aggregate JC-10 red fluorescence (λ_ex_ = 540/λ_em_ = 590 nm) were measured using a fluorescence spectrophotometer (SpectraMax M2 microplate reader, Molecular Devices, Sunnyvale, CA, USA), and the ratio of red to green fluorescence was calculated.

For monitoring apoptosis, cells were plated on 18 mm coverslips. After the end of treatment, the fixed cells were reacted with JC-10 solution for 1 h at 37 °C according to the manufacturer’s instructions, and visualized by fluorescence microscopy (ZEISS, Jena, Germany).

### 2.11. ATP Detection Assay

Cellular ATP levels were measured using a luminescent ATP detection assay kit (Abcam, Cambridge, UK) based on firefly luciferase in accordance with the manufacturer’s instructions. Briefly, cells were seeded into a 96-well white plate. At the end of treatment, the cells were lysed with detergent and reacted with substrate solution on a shaker at room temperature in the dark. The plate was dark-adapted by covering it for 10 min. The luminescence was measured using a luminescence spectrophotometer (SpectraMax M2 microplate reader, Molecular Devices, Sunnyvale, CA, USA).

### 2.12. Western Blot Analysis

Twenty micrograms of total protein was separated by 12.5% sodium dodecyl sulfate–polyacrylamide gel electrophoresis (SDS-PAGE) and transferred to a polyvinylidene fluoride (PVDF) membrane (Millipore, Billerica, MA, USA) for immunoblot analysis using the following primary antibodies: GRP78 (1:1000), GADD153 (1:1000), Bax (1:2000), Bcl-2 (1:1000), cytochrome c (1:500), caspase-9 (1:1000), caspase-3 (1:1000), and GAPDH (1:1000). The primary antibodies were purchased from Cell Signaling Technology (Danvers, MA, USA), except for the Bcl-2 and GAPDH antibodies, which were purchased from Santa Cruz Biotechnology (Dallas, TX, USA). The PVDF membranes were incubated overnight at 4 °C with the primary antibodies, and subsequently incubated for 1 h at room temperature with a horseradish peroxidase (HRP)-conjugated secondary antibody, goat anti-rabbit immunoglobulin G (IgG) or horse anti-mouse IgG (Cell Signaling Technologies, Danvers, MA, USA). The target protein bands were detected using a chemiluminescence image analyzer (CAS-400SM, Davinch-K, Seoul, Korea) and quantified using the ImageJ software (National Institutes of Health, Bethesda, MD, USA). The protein levels were normalized to the GAPDH protein levels.

### 2.13. Statistical Analysis

The results are presented as means ± standard deviations (SDs). All statistical analyses were performed with one-way ANOVA using the JMP 5 software (SAS Campus Drive, Cary, NC, USA). The values were considered significantly different at *p* < 0.01 or 0.05. All experiments were performed a minimum of three times.

## 3. Results

### 3.1. WEST Alleviates Corticosterone-Induced Injury in PC12 Cells

**Figure 1:**
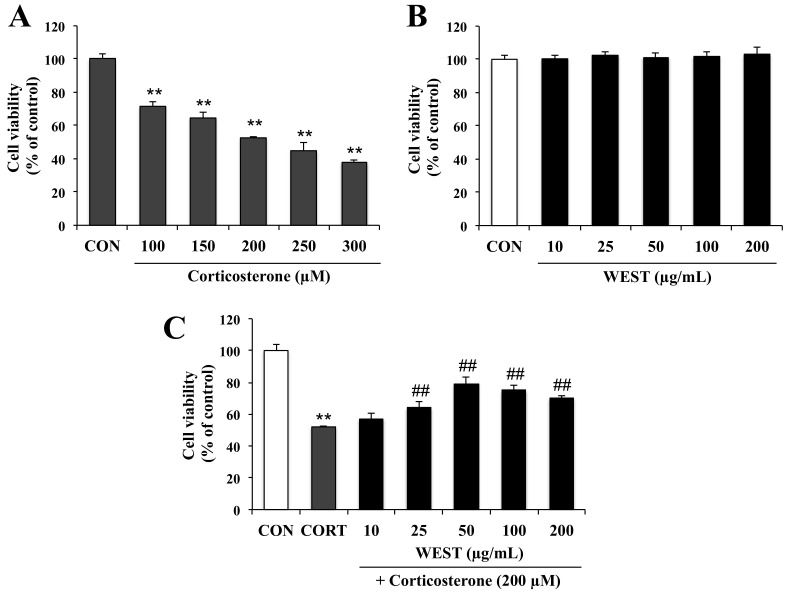
Effect of water extract of *S. takesimense* (WEST) pretreatment on cell viability in corticosterone-treated PC12 cells measured using the EZ-cytox assay. (**A**) Concentration-dependent cell viability of corticosterone-treated PC12 cells; (**B**) Concentration-dependent cell viability of WEST-treated PC12 cells; (**C**) Cell viability of PC12 cells pretreated with WEST at the concentrations shown prior to 200 µM corticosterone treatment compared to cells treated with 200 µM corticosterone only and non-treated control cells. CON, non-treated control; CORT, corticosterone treatment; WEST, water extract of *S.takesimense*. Bars represent mean ± SD. ** *p* < 0.01 compared with CON group; ## *p* < 0.01 compared with CORT group.

To determine the appropriate concentration of corticosterone for achieving cell damage, PC12 cells were incubated for 24 h with 100, 150, 200, 250, and 300 µM corticosterone. Cell viability was decreased in a corticosterone-concentration-dependent manner by 72, 65, 52, 45, and 38%, respectively (Figure 1A). Treatment with 200 µM corticosterone decreased survival to about half that of the CON group; this concentration was therefore used in subsequent experiments to induce cell death.

To assess the cytotoxicity of WEST in PC12 cells, the cells were treated with various concentrations (10, 25, 50, 100, and 200 µg/mL) of WEST for 24 h. As shown in Figure 1B, WEST had no intrinsic toxic effect on the cells. Subsequently, the cells were treated with 200 µM corticosterone at different concentrations of WEST, as mentioned above, to determine the cytoprotective effects of WEST against corticosterone-induced damage in PC12 cells. As shown in Figure 1C, exposure of PC12 cells to corticosterone led to a significant decrease in viability compared with that in the CON group. The cell survival rate of the corticosterone-treated cells was 52% that of the controls, while pretreatment with 10, 25, 50, 100, and 200 µg/mL of WEST increased the survival of PC12 cells in a dose-dependent manner by 57, 63, 81, 75, and 70%, respectively. Treatment with 50 µg/mL of WEST resulted in the maximal protective effects against corticosterone injury. Thus, in order to further study the cytoprotective effect and possible mechanism of action of WEST against corticosterone in PC12 cells, we used 200 µM corticosterone for a negative group (CORT group) and 50 µg/mL of WEST with 200 µM corticosterone as the sample-treatment group (WEST + CORT group).

**Figure 2:**
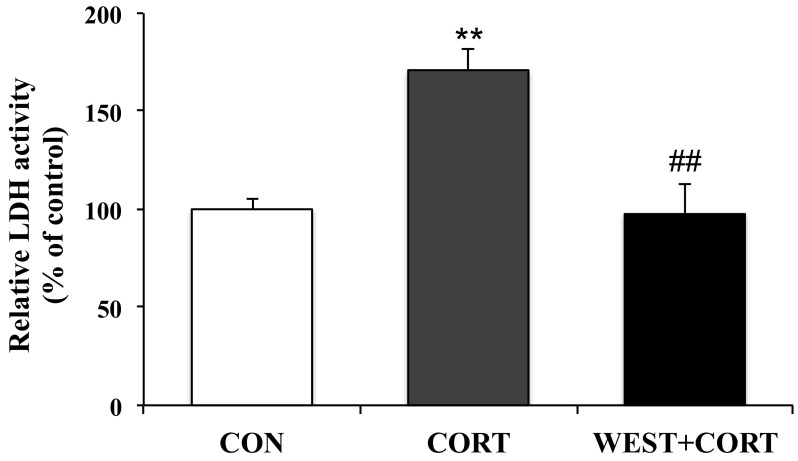
Effect of WEST pretreatment on lactate dehydrogenase (LDH) leakage in corticosterone-treated PC12 cells. CON, non-treated control; CORT, 200 µM corticosterone treatment; WEST + CORT, 50 µg/mL WEST pretreatment plus 200 µM corticosterone treatment. Bars represent mean ± SD. ** *p* < 0.01 compared with CON group; ## *p* < 0.01 compared with CORT group.

Since the LDH released from cells is widely used as a cellular damage marker, LDH leakage was measured in the culture medium to assess PC12 cell injury. LDH release from cells significantly increased in the CORT group compared to that in the CON group; the percentage of LDH leakage increased from 100% (control) to 171 ± 10%. However, the WEST + CORT group showed decreased LDH leakage, at a rate of 98 ± 15% (Figure 2). Meanwhile, LDH leakage with WEST treatment alone was similar to that in the CON group (Figure S1). These data indicate that WEST pretreatment could effectively prevent corticosterone-induced injury in PC12 cells.

### 3.2. WEST Inhibits Apoptosis Induced by Corticosterone in PC12 Cells

**Figure 3:**
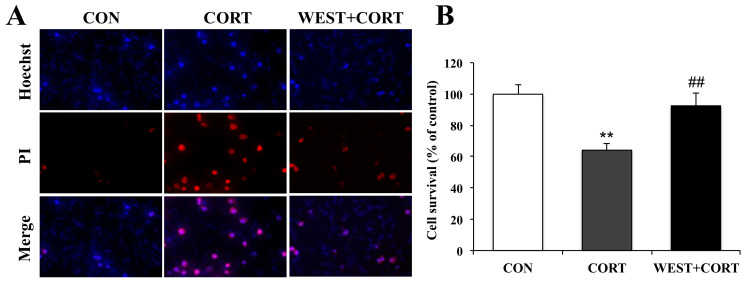
Effects of WEST pretreatment on cell survival in corticosterone-treated PC12 cells assessed by Hoechst 33342 and PI staining. (**A**) Photomicrographs showing double staining with Hoechst 33342 and PI, representative images of PI-positive cells (red, middle row), Hoechst counterstaining (blue, top row), and merged image (bottom row). (**B**) Cell survival was detected by manually counting the cells, as in (**A**). CON, non-treated control; CORT, 200 µM corticosterone treatment; WEST + CORT, 50 µg/mL WEST pretreatment plus 200 µM corticosterone treatment. Bars represent mean ± SD. ** *p* < 0.01 compared with CON group; ## *p* < 0.01 compared with CORT group.

We next measured the protective effects of WEST on corticosterone-induced apoptosis by Hoechst 33342 and PI double staining, which is extensively used for the fluorescence imaging analysis of the various stages of apoptosis. As shown in the microphotographs of Figure 3A, the number of PI-positive cells was increased in the CORT group, and the cell survival rate decreased significantly to 64 ± 4% as compared with the CON group (Figure 3B). By contrast, apoptotic nuclei were decreased in the WEST + CORT group; the survival rate increased to 92 ± 8% of the control (Figure 3A,B), while WEST treatment alone did not change the apoptosis rate of PC12 cells (Figure S2).

**Figure 4:**
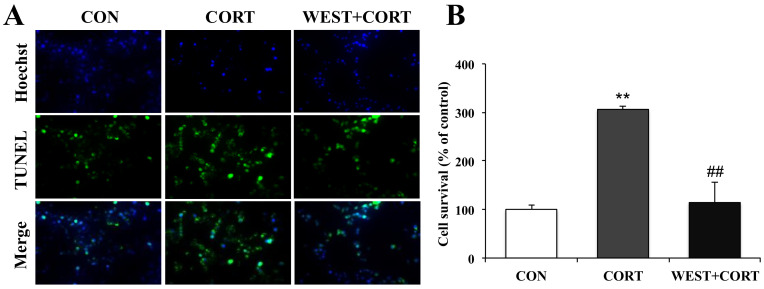
Effects of WEST pretreatment on the viability of corticosterone-treated PC12 cells according to TUNEL staining. (**A**) Representative image showing TUNEL-positive cells (green, middle row), Hoechst counterstaining (blue, top row), and merged image (bottom row). (**B**) Apoptotic percentage was calculated by manually counting the cells, as in (**A**). CON, non-treated control; CORT, 200 µM corticosterone treatment; WEST + CORT, 50 µg/mL WEST pretreatment plus 200 µM corticosterone treatment. Bars represent mean ± SD. ** *p* < 0.01 compared with CON group; ## *p* < 0.01 compared with CORT group.

To further confirm the protective role of WEST in corticosterone-induced apoptosis in PC12 cells, we also observed the nuclear morphology by TUNEL staining, which is widely used for DNA fragmentation detection. As shown in Figure 4A, very few green fluorescent TUNEL-positive cells were seen in the CON group, while their presence was significantly increased in the CORT group, where the incidence of TUNEL-labeled cells markedly increased from 100% (control) to 306 ± 10% (Figure 4B). By contrast, the presence of TUNEL-labeled cells was dramatically decreased in the WEST + CORT group, and the apoptotic rate was 114 ± 38% compared to that in the CON group (Figure 4A,B). WEST treatment alone had no effect on the apoptosis rate of PC12 cells (Figure S3).

These data indicate that WEST pretreatment could inhibit apoptosis by effectively reducing apoptotic nuclei in corticosterone-induced apoptotic PC12 cells.

### 3.3. WEST Prevents Corticosterone-Induced ER Stress in PC12 Cells

To determine whether corticosterone-induced apoptosis in PC12 cells was related to ER stress, we analyzed the protein expression levels of ER stress biomarkers, GRP78 and GADD153, and changes in the intracellular Ca^2+^ concentration as an indicator of apoptosis in the ER.

**Figure 5:**
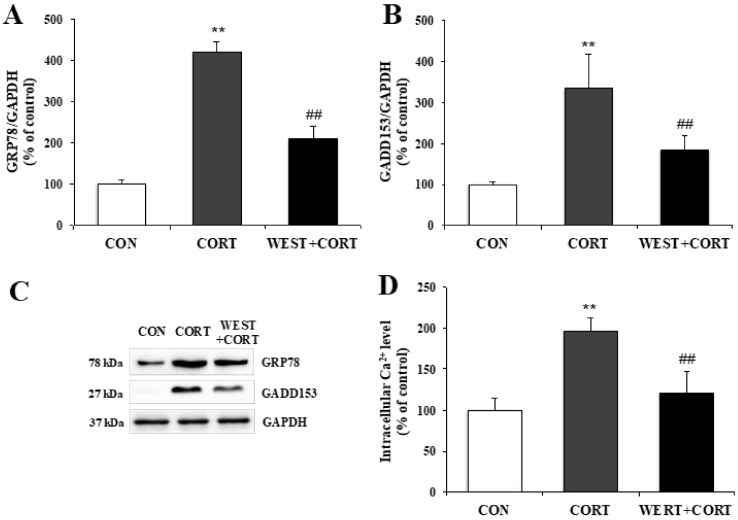
Effect of WEST pretreatment on corticosterone-induced endoplasmic reticulum (ER) stress in PC12 cells. Protein expression of ER stress biomarkers (**A**) glucose-regulated protein 78 (GRP78) and (**B**) growth arrest- and DNA damage-inducible gene (GADD)153 in corticosterone-treated PC12 cells pretreated with WEST versus corticosterone-treated or non-treated control cells, normalized to glyceraldehyde 3-phosphate dehydrogenase GAPDH protein expression. (**C**) Western blot image of GRP78, GADD153, and GAPDH protein expression. (**D**) Ca^2+^ concentration in corticosterone-treated PC12 cells pretreated with WEST versus corticosterone-treated or non-treated control cells. CON, non-treated control; CORT, 200 µM corticosterone-treatment; WEST + CORT, 50 µg/mL WEST pretreatment plus 200 µM corticosterone treatment. Bars represent mean ± SD. ** *p* < 0.01 compared with CON group; ## *p* < 0.01 compared with CORT group. kDa; kilodalton.

As shown in Figure 5A–C, the GRP78 and GADD153 protein levels significantly increased in the CORT group relative to the CON group, and decreased in the WEST + CORT group compared to those in the CORT group. These results demonstrate that ER stress markers were induced by corticosterone treatment and downregulated by pretreatment with WEST.

As shown in Figure 5D, the Ca^2+^ concentration markedly increased in the CORT group to 196 ± 16%, compared to that in the CON group. By contrast, in the WEST + CORT group, the corticosterone-induced Ca^2+^ elevation was significantly attenuated compared to that in the CORT group, at 121 ± 26% (Figure 5D). Meanwhile, the Ca^2+^ concentration with WEST treatment alone was similar to that in the CON group (Figure S4). These results show that corticosterone treatment increased the intracellular Ca^2+^ concentration, whereas WEST pretreatment decreased corticosterone-induced intracellular Ca^2+^ elevation, in PC12 cells.

### 3.4. WEST Protects PC12 Cells against Mitochondrial Dysfunction Following Cell Damage

During apoptosis, the excessive accumulation of Ca^2+^ in mitochondria induces mitochondrial dysfunction due to the opening of the mPTP and subsequent collapse of the MMP. We next measured the mPTP, MMP, intracellular ATP, and ROS levels to determine whether WEST could improve mitochondrial dysfunction caused by cell damage.

**Figure 6:**
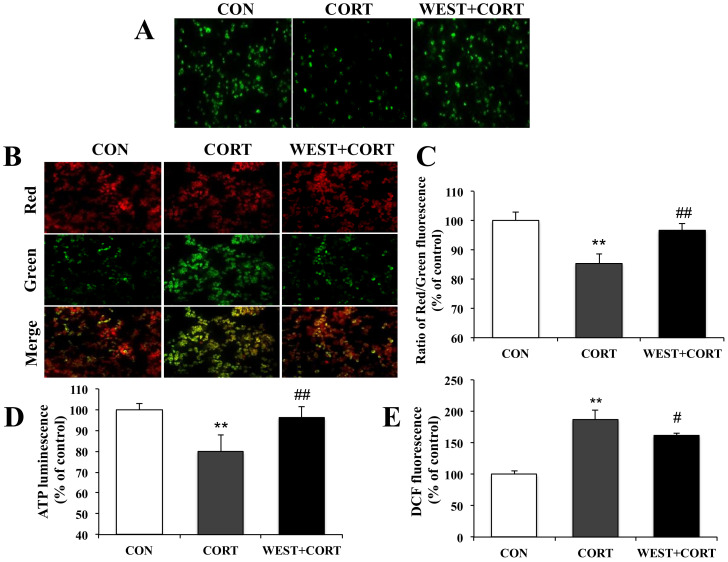
Effect of WEST pretreatment against corticosterone-induced mitochondrial dysfunction in PC12 cells. (**A**) Effects of WEST pretreatment on the opening of mitochondrial permeability transition pores (mPTPs). (**B**) Effects of WEST pretreatment on mitochondrial membrane potential (MMP) collapse. (**C**) The fluorescence intensity was measured using a fluorescence spectrophotometer after JC-10 staining, as in (**B**). (**D**) Effect of WEST pretreatment on intracellular ATP levels. (**E**) Effect of WEST pretreatment on intracellular reactive oxygen species (ROS) levels. The 2′,7′-dichlorofluorescein diacetate (DCF) fluorescence reflects the ROS level. CON, non-treated control; CORT, 200 µM corticosterone treatment; WEST + CORT, 50 µg/mL WEST pretreatment plus 200 µM corticosterone treatment. Bars represent mean ± SD. ** *p* < 0.01 compared with CON group; ## *p* < 0.01 compared with CORT group; # *p* < 0.05 compared with CORT group.

We first assayed the mPTP opening. As shown for the CON group in Figure 6A, we could monitor the distribution of intact mPTP as green fluorescence in healthy cells. By contrast, as shown in the CORT group, when mPTPs were abnormally opened by corticosterone treatment, the fluorescence distribution was decreased compared to that in the CON group, but the intensive fluorescence was seen again upon pretreatment with WEST (Figure 6A). Treatment with WEST alone did not influence the green fluorescence (Figure S5). The results indicated that WEST pretreatment induced the closure of mPTPs opened by corticosterone.

We next determined the changes in MMP. As shown in Figure 6B, the green fluorescence was increased in the CORT group relative to that in the CON group, while the green fluorescence was attenuated in the WEST + CORT group compared with that in the CORT group. The fluorescence intensity was measured, and the ratio of red to green fluorescence was calculated. The red/green fluorescence ratio of the CORT group decreased to 85 ± 3% of that of the control, while it was 97 ± 2% of the control in the WEST + CORT group (Figure 6C). These results indicate that the exposure of PC12 cells to corticosterone disrupted the integrity of the MMP, while WEST pretreatment prevented the corticosterone-induced disruption of the MMP.

We further measured intracellular ATP and ROS levels. As shown for the CORT group in Figure 6D, the intracellular ATP levels were significantly decreased, by corticosterone treatment, to 79 ± 7% compared to those in the CON group. In the WEST + CORT group, the ATP levels were increased compared to those in the CORT group, to 96 ± 5% of the CON group’s. These results show that corticosterone decreased intracellular ATP levels, whereas WEST pretreatment prevented the ATP levels from being reduced by corticosterone in the cells. Intracellular ROS levels were remarkably increased in the CORT group compared to those in the CON group, and the ROS levels increased from 100% (control) to 187 ± 14% (Figure 6E). By contrast, the WEST + CORT group showed a decrease in ROS levels compared with the CORT group, with levels of 162 ± 4% of the control (Figure 6E). These results indicate that ROS production is induced when cells are exposed to corticosterone and that the ROS produced by corticosterone are weakened in cells pretreated with WEST.

### 3.5. WEST Modulates the Expression of Apoptosis-Related Proteins in Corticosterone-Treated PC12 Cells

To examine the mechanisms of WEST-mediated changes in the induction of apoptosis in corticosterone-treated PC12 cells, we analyzed the expression of apoptosis-related proteins, Bax, Bcl-2, cytochrome c, caspase-9, and caspase-3, using Western blotting.

**Figure 7:**
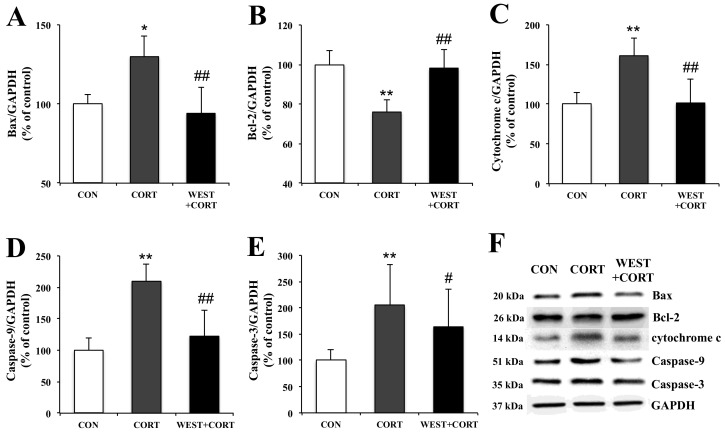
Effects of WEST pretreatment on the expression of apoptosis-related proteins in corticosterone-treated PC12 cells. Protein expression of (**A**) Bax, (**B**) Bcl-2, (**C**) cytochrome c, (**D**) caspase-9, and (**E**) caspase-3 in PC12 cells pretreated with WEST and treated with corticosterone compared to corticosterone-treated and non-treated cells. The protein levels were normalized to the GAPDH protein level. (**F**) Western blot image of Bax, Bcl-2, cytochrome c, caspase-9, caspase-3, and GAPDH protein expression. CON, non-treated control; CORT, 200 µM corticosterone treatment; WEST+CORT, 50 µg/mL WEST pretreatment plus 200 µM corticosterone treatment. Bars represent mean ± SD. ** *p* < 0.01 compared with CON group; * *p* < 0.05 compared with CON group; ## *p* < 0.01 compared with CORT group; # *p* < 0.05 compared with CORT group. kDa; kilodalton.

In the changes in Bax and Bcl-2 expression, the Bax expression level was upregulated by corticosterone treatment, but the Bcl-2 expression level was downregulated under the same conditions (Figure 7A,B). However, pretreatment with WEST reversed these effects (Figure 7A,B).

Mitochondrial apoptotic pathway-related proteins, cytochrome c, caspase-9, and caspase-3, were also detected. As shown in Figure 7C–E, the expression levels of the cytochrome c, caspase-9, and caspase-3 proteins were markedly increased in the CORT group as compared with the control group. However, their expression levels were significantly decreased in the WEST + CORT groups. The expression of these proteins in the WEST-treated cells was similar to that in the CON group (Figure S6). These results show that mitochondrial apoptosis was induced upon corticosterone treatment and prevented by pretreatment with WEST.

## 4. Discussion

The ER plays a central role in the biosynthesis of proteins and lipids, and in the storage of calcium in cells, while mitochondria are organelles central to the production of ATP as well as to the synthesis and processing of various metabolites, and regulation of cell death [28,29]. These organelles play essential roles in sensing and responding to cell stress, and their interactions affect the functional organization of organelles and ultimately regulate cell survival [29]. ER stress is a crucial trigger in the apoptotic process, which can lead to mitochondrial dysfunction via various mechanisms, inducing apoptosis [30,31].

It has been reported that the expression of GRP78 and GADD153 is significantly enhanced during ER stress [32,33]. GRP78 is an ER chaperone, important for ER function as a master regulator of the unfolded protein response (UPR) [34]. GADD153 is a transcription factor encoded by the DNA damage inducible transcript 3 (*DDIT3*) gene, which is highly upregulated during ER stress [35].

Our study demonstrated that the ER stress markers GRP78 and GADD153 were significantly increased in corticosterone-stressed PC12 cells. However, the expression of these proteins was reduced in PC12 cells pretreated with WEST. This study also found that the concentration of intracellular Ca^2+^, another major indicator of ER stress, was upregulated in corticosterone-treated PC12 cells but downregulated in cells pretreated with WEST. These results indicate that the neuroprotective effect of WEST against corticosterone-induced apoptosis was mediated by the inhibition of ER stress.

Ca^2+^ signaling is crucial for physiological and functional interactions between the ER and mitochondria [36]. The unique juxtaposition of these organelles plays a crucial role in the pathogenesis of metabolic diseases [37]. During chronic ER stress, a resistive response occurs, and abnormal calcium signals are transmitted from the ER to the mitochondria, leading to cell death [38].

The overloading of Ca^2+^ released from the ER due to intracellular stress induces mitochondrial dysfunction by inducing the depolarization of the inner mitochondrial membrane (IMM), mPTP opening, and ROS generation, ultimately promoting the activation of the caspase-regulated apoptosis pathway [39,40]. The magnitude and outcome of Ca^2+^ responses during apoptosis can be regulated by various Bcl-2 family members [29,41]. The Bcl-2 family members contain several homologs, including anti-apoptotic proteins and pro-apoptotic proteins [42]. The anti-apoptotic proteins Bcl-2 and Bcl-XL inhibit Ca^2+^ delivery, while the pro-apoptotic proteins Bax and Bak stimulate Ca^2+^ mobilization [43,44,45]. The resulting balance between anti- and pro-apoptotic members has been proven to influence the mitochondrial apoptosis pathway. Nutt et al. reported that the overexpression of Bax and Bak promotes apoptosis by increasing Ca^2+^ levels [43]. In the current study, the intracellular Ca^2+^ concentration was increased, the protein expression of Bax was enhanced, and that of Bcl-2 was attenuated in PC12 cells treated with corticosterone. These results are consistent with those of other studies [8,26,46]. By contrast, we confirmed that these effects were reversed in PC12 cells pretreated with WEST.

The overaccumulation of Ca^2+^ in mitochondria causes the transient depolarization of the IMM [47], leading to the opening of mPTPs, which are voltage-dependent, high-conductance channels [48]. The uncontrolled opening of mPTPs leads to MMP collapse, and several pro-apoptotic proteins, such as cytochrome c and apoptosis inducing factor (AIF), are released into the cytosol from the intermembrane space through mPTPs, where they modulate the final steps of the apoptotic cascade [49,50]. Thus, the IMM potential is also essential for signaling, especially in cell survival decisions and mitophagy [29]. In the present study, corticosterone treatment led to abnormal mPTP opening and MMP collapse in PC12 cells. By contrast, pretreatment with WEST inhibited mPTP opening and stabilized the MMP.

The IMM includes the electron transport chain (ETC; complexes I–IV) of the oxidative phosphorylation system, and the electrons are passed from the electron donors to electron acceptors through the ETC [51]. This electron transfer couples with the transfer of protons across the IMM, creating an electrochemical proton gradient that drives the synthesis of ATP by F_1_-F_0_-ATPase in the IMM [48]. Most of the proton motive force is contained in the MMP; thus, decreasing the MMP reduces the amount of free energy available to produce ATP [48]. In this study, we confirmed that the generation of ATP decreased with the loss of MMP upon corticosterone treatment and found that these effects were reversed in PC12 cells pretreated with WEST. Thus, the stability of the MMP is vital for maintaining the function of mitochondria, and these findings prove that WEST prevents the mitochondrial dysfunction induced by corticosterone.

**Figure 8:**
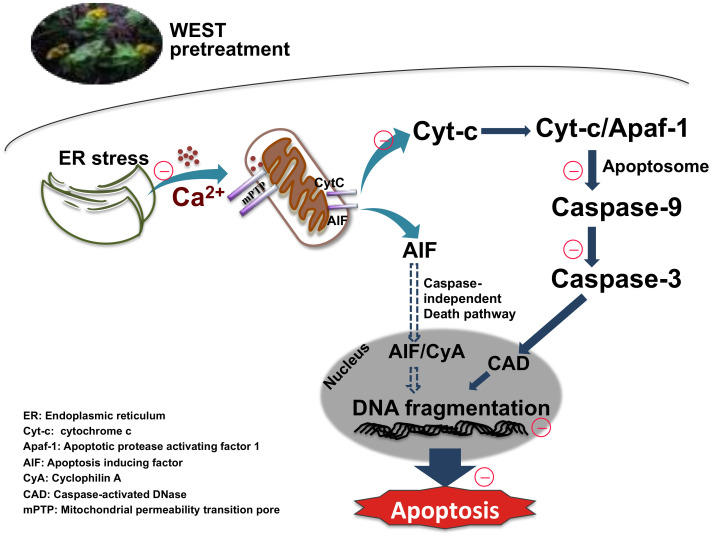
*Sedum takesimense* protects PC12 cells against corticosterone-induced neurotoxicity through the inhibition of the apoptotic pathway during ER stress and mitochondrial dysfunction.

We next assessed the expression of the mitochondrial apoptotic pathway-related proteins cytochrome c, caspase-9, and caspase-3. Corticosterone treatment upregulated cytochrome c, caspase-9, and caspase-3 protein expression, consistent with other studies [8,26,46,52]. By contrast, pretreatment with WEST downregulated the expression of these proteins. Our data demonstrate that WEST markedly attenuated the overexpression of cytochrome c, caspase-9, and caspase-3 induced by corticosterone. In the final step of apoptosis, caspase-3 cleaves the inhibitor of caspase-activated DNase (ICAD), which leads to the activation of caspase-activated DNase (CAD), generating DNA fragments in the nucleus [53]. We determined that DNA fragmentation was increased by corticosterone-induced apoptosis in PC12 cells pretreated with WEST. We confirmed that WEST can inhibit apoptosis by effectively reducing apoptotic nuclei (Figure 8).

We also measured intracellular ROS levels in corticosterone-induced injury. Several studies have shown that glucocorticoid treatment alters the activity of antioxidant enzymes and thus increases the ROS level [54,55]. Excessively elevated ROS levels alter the cellular redox balance and cause damage to major macromolecules [54]. In particular, neuronal cells are susceptible to ROS-induced damage, partly due to their low levels of antioxidant enzymes, resulting in a deficient antioxidant defense system [54]. In the present study, PC12 cells exposed to corticosterone significantly increased the intracellular ROS level, consistent with the results of other studies [52,54]. However, pretreatment with WEST reduced ROS levels.

Several studies have reported that *S. takesimense* has antioxidant constituents [24,56]; our study also showed that the ROS increased by corticosterone were reduced by treatment with WEST. As many studies have shown that ER stress and the mitochondrial apoptosis pathway are attenuated by antioxidants [57,58,59], the WEST-mediated cytoprotective effect might have been partly due to the antioxidant effects that inhibit apoptosis.

We have demonstrated that WEST exerts a neuroprotective effect by inhibiting the apoptotic pathway involved in corticosterone-induced ER stress and mitochondrial dysfunction. Although the cellular mechanisms of action of WEST have not yet been fully elucidated, our results provide novel evidence that WEST reduces neuronal damage due to the neurotoxicity caused by chronic stress. However, the use of plant extracts is limited by various factors. Firstly, it is difficult to define the composition and role of the active molecules. In addition, it is difficult to reproduce the action of the extract, due to the variability in its composition, which is caused by various factors (season, humidity, temperature, etc.). Considering these limitations, further studies on the neuroprotective effects of WEST using animal models and clinical samples are warranted. Further studies may be used to develop new therapeutics that protect against diseases caused by neurological dysfunction.

## Figures and Tables

**Table 1:** Compounds isolated from *S. takesimense* [24].

Compounds	
ferulic acid caffeic acid gallic acid methyl gallate myricetin quercetin luteolin rhodalin	rhodalidin arbutin 2,6-di-*O*-galloylarbutin luteolin-7-*O*-β-D-glucoside gossypetin-8-*O*-β-D-xylopyranoside 1-(4-hydroxyphenyl)-2-(3,5-dihydroxyphenyl)-2-hydroxyethanone

## Supplementary Materials

The following are available online at https://www.mdpi.com/2072-6643/12/12/3713/s1, Figure S1: Effect of WEST on LDH leakage in PC12 cells. Figure S2: Effects of WEST treatment on cell survival in PC12 cells, as assessed with Hoechst 33342 and PI staining. Figure S3: Effects of WEST treatment on the viability of PC12 cells, as assessed by TUNEL staining. Figure S4: Effect of WEST treatment on intracellular Ca^2+^ concentration of PC12 cells. Figure S5: Effect of WEST treatment on (A) the mPTP opening, (B) MMP collapse, (C) the intracellular ATP levels, (D) the intracellular ROS levels. Figure S6: Effects of WEST treatment on the expression of apoptosis-related proteins in PC12 cells.
