# Supplementary material for: Sedum takesimense Protects PC12 Cells against Corticosterone-Induced Neurotoxicity by Inhibiting Neural Apoptosis
Source: Nutrients. 2020 Nov 30;12(12):3713. doi: 10.3390/nu12123713 (PMC7759901; doi:10.3390/nu12123713)
Supplement: Supplementary file 1 [file nutrients-12-03713-s001.pdf]

Supplementary material

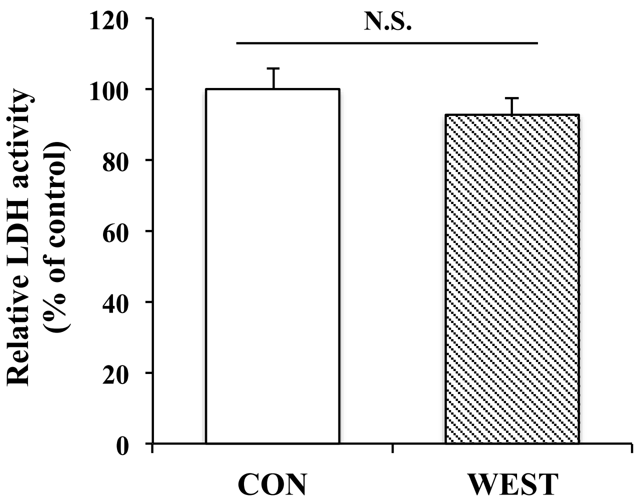

Figure S1. Effect of WEST on LDH leakage in PC12 cells. CON, non-treated control; WEST, 50 μg/mL WEST-treatment. Bars represent mean ± SD. N.S., not significant.

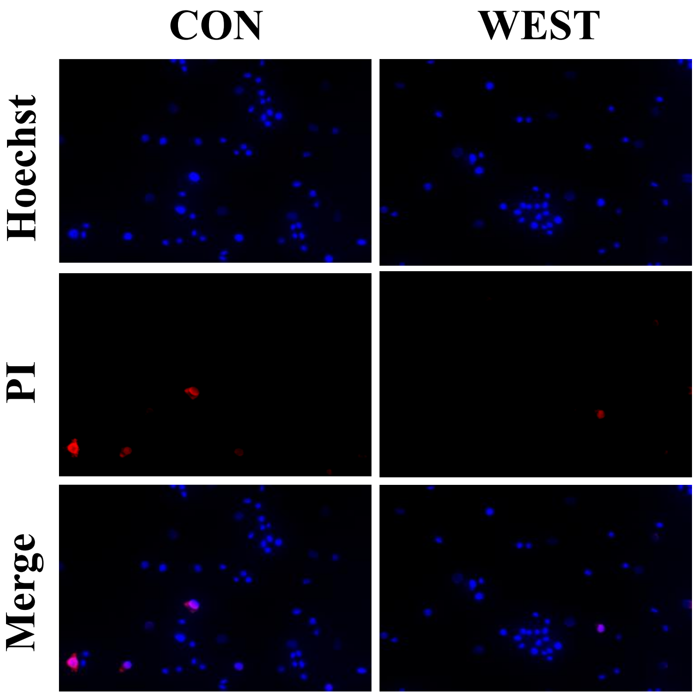

Figure S2. Effects of WEST treatment on cell survival in PC12 cells, as assessed with Hoechst 33342 and PI staining. Photomicrographs showing double staining with Hoechst 33342 and PI, with representative images of PI-positive cells (red, middle row) and Hoechst counterstaining (blue, top row) and the merged images (bottom row). CON, non-treated control; WEST, 50 μg/mL WEST-treatment. Bars represent mean ± SD. N.S., not significant.

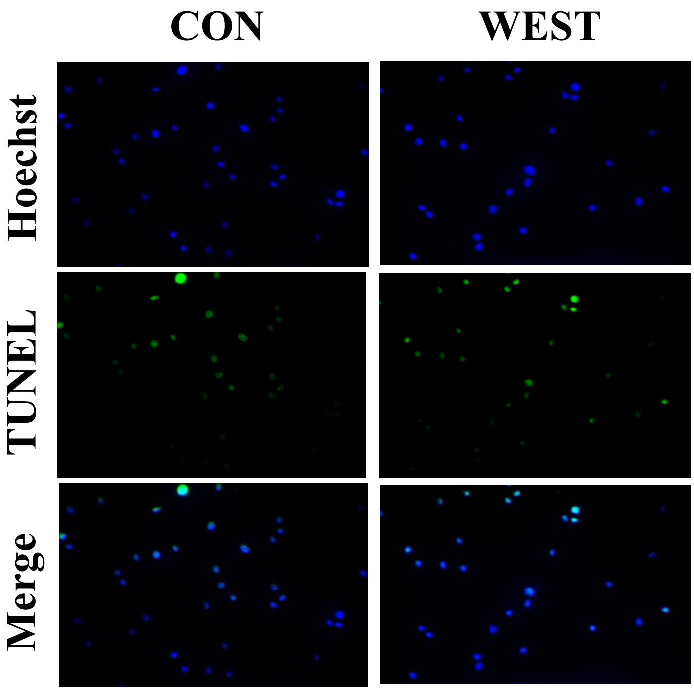

Figure S3. Effects of WEST treatment on the viability of PC12 cells, as assessed by TUNEL staining. Representative images showing TUNEL-positive cells (green, middle row), Hoechst counterstaining (blue, top row), and merged image (bottom row). CON, non-treated control; WEST, 50 μg/mL WEST-treatment. Bars represent mean ± SD. N.S., not significant.

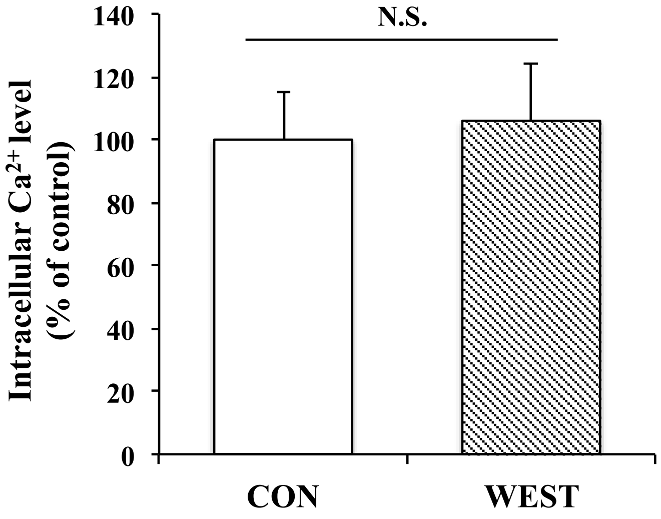

Figure S4. Effect of WEST treatment on intracellular Ca^{2+} concentration of PC12 cells. CON, non-treated control; WEST, 50 µg/mL WEST-treatment. Bars represent mean ± SD. N.S., not significant.

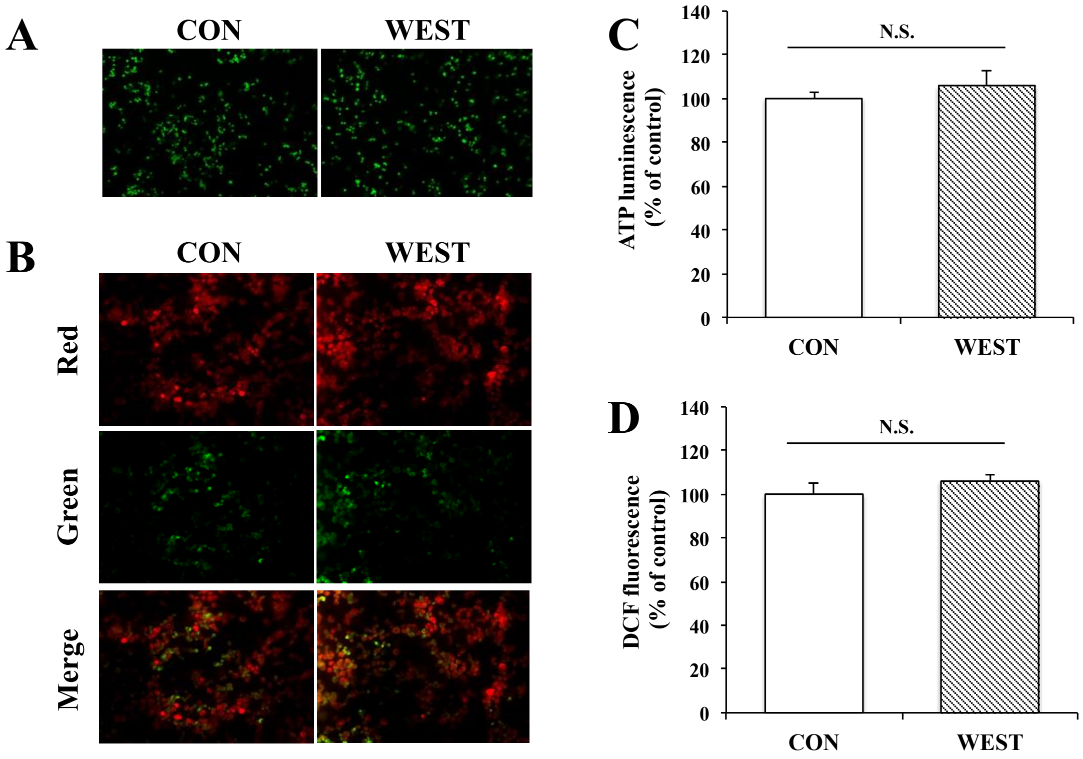

Figure S5. Effect of WEST treatment on **(A)** the mPTP opening, **(B)** MMP collapse, **(C)** the intracellular ATP levels, **(D)** the intracellular ROS levels. DCF fluorescence reflects the ROS level. CON, non-treated control; WEST, 50 µg/mL WEST-treatment. Bars represent mean ± SD. N.S., not significant.

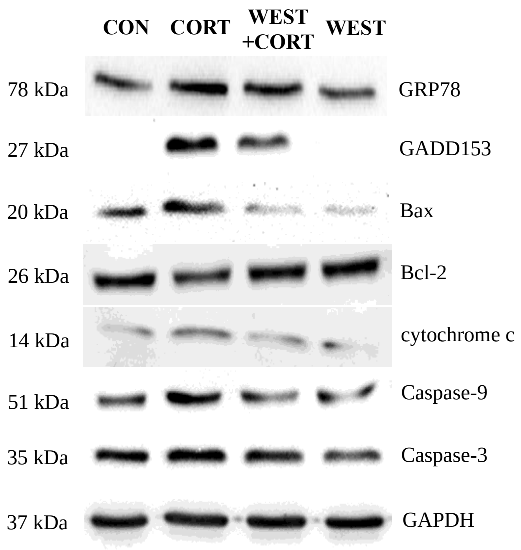

Figure S6. Effects of WEST treatment on the expression of apoptosis-related proteins in PC12 cells. Western blots showing the expression of GRP78, GADD153, Bax, Bcl-2, cytochrome c, Caspase-9, Caspase-3, and GAPDH. CON, non-treated control; CORT, 200 μM corticosterone-treatment; WEST+CORT, 50 μg/mL WEST-pretreatment plus 200 μM corticosterone-treatment; WEST, 50 μg/mL WEST-treatment.
